# Announcement Extraordinary

**Published:** 1896-09

**Authors:** 


### Editorial.

### ANNOUNCEMENT EXTRAORDINARY.

THE subscription price of the SOUTHERN MEDICAL RECORD has been reduced to $1.00 per year. Increase in circulation and the financial depression that seems to have fallen so heavily upon our subscribers have induced us to make the change. There will be no falling off whatever in the size or literary tone of the journal. The terms are, payable in advance, and all subscribers, old and new, are entitled to the new rate.

